# The Aβ42 Peptide and IAPP Physically Interact in a Yeast-Based Assay

**DOI:** 10.3390/ijms241814122

**Published:** 2023-09-15

**Authors:** Daniel V. Kachkin, Veronika V. Lashkul, Natalia A. Gorsheneva, Sergey A. Fedotov, Maria S. Rubel, Yury O. Chernoff, Aleksandr A. Rubel

**Affiliations:** 1 Laboratory of Amyloid Biology, St. Petersburg State University, St. Petersburg 199034, Russia; daniil.kachkin@uk-erlangen.de (D.V.K.); serg900@yandex.ru (S.A.F.); 2 Pavlov Institute of Physiology, Russian Academy of Sciences, St. Petersburg 199034, Russia; 3 Laboratory of DNA-Nanosensor Diagnostics, SCAMT Institute, ITMO University, St. Petersburg 191002, Russia; rubel@scamt-itmo.ru; 4 School of Biological Sciences, Georgia Institute of Technology, Atlanta, GA 30332, USA; yury.chernoff@biology.gatech.edu; 5 Pediatric Research and Clinical Center for Infectious Diseases, Department of Medical Microbiology and Molecular Epidemiology, St. Petersburg 197022, Russia

**Keywords:** amyloidosis, Alzheimer’s disease, type 2 diabetes mellitus, protein–protein interactions, protein aggregation, amyloid beta, PrP, IAPP, FRET

## Abstract

Numerous studies have demonstrated that people with type 2 diabetes mellitus (associated with IAPP peptide aggregation) show an increased incidence of Alzheimer’s disease (associated with Aβ aggregation), but the mechanism responsible for this correlation is presently unknown. Here, we applied a yeast-based model to study the interactions of IAPP with PrP (associated with TSEs) and with the Aβ42 peptide. We demonstrated that fluorescently tagged IAPP forms detergent-resistant aggregates in yeast cells. Using the FRET approach, we showed that IAPP and Aβ aggregates co-localize and physically interact in yeast cells. We also showed that this interaction is specific and that there is no interaction between IAPP and PrP in the yeast system. Our data confirmed a direct physical interaction between IAPP and Aβ42 aggregates in a living cell. Based on these findings, we hypothesize that this interaction may play a crucial role in seeding Aβ42 aggregation in T2DM patients, thereby promoting the development of AD.

## 1. Introduction

Protein misfolding disorders (PMDs) are a broad heterogeneous group of diseases characterized by the formation and accumulation of misfolded protein aggregates in various tissues and organs. These aggregates, known as amyloids, are fibrous non-covalent cross-β polymers formed by disease-specific misfolded proteins or peptides [1]. More than 40 amyloidogenic proteins and peptides are linked to PMDs [2] and cause approximately 70 different known forms of amyloidosis in humans [2]. These include tau protein and amyloid-beta peptide (Aβ) in Alzheimer’s disease (AD) [3], huntingtin (Htt) in Huntington’s disease (HD) [4], α-synuclein in Parkinson’s disease (PD) [5], islet amyloid polypeptide (IAPP), or amylin in type 2 diabetes mellitus (T2DM) [6], TDP-43 in amyotrophic lateral sclerosis (ALS) [7] and frontotemporal dementia (FTD) [7], and prion protein (PrP) in transmissible spongiform encephalopathies or TSEs [8].

Moreover, histological studies have demonstrated that in some cases, multiple amyloid proteins can be found within a deposit or in close proximity to it [9]. For instance, it is common to find aggregates of the Aβ peptide and tau protein in AD brain tissues with some accumulation of α-synuclein and TDP-43 associated with PD brain (see reviews [10,11]). PrP can also be a component of amyloid plaques in the brains of AD and PD patients [12], while Aβ can be found within prion aggregates in TSE patients [13]. Similarly, IAPP can aggregate in the brain of patients with AD [14] and PD [15], while aggregates of Aβ and tau protein can be found in the pancreas of T2DM patients [16]. The mechanisms responsible for the co-aggregation of different amyloidogenic proteins are not well understood, but it is believed that the aggregation of one protein may induce the misfolding and aggregation of another protein (the phenomenon known as “cross-seeding”) (see [17]). Thus, protein aggregates or oligomers of one amyloidogenic protein, in principle, can induce not only disease-specific damage but can also lead to other pathologies by cross-seeding an unrelated protein.

T2DM and AD are two of the most prevalent protein misfolding disorders worldwide, and numerous studies have shown an interconnection between those two impairments. Many epidemiological studies have revealed a significantly higher risk for the development of AD in patients affected by T2DM, but the exact molecular mechanisms responsible for this association are presently unknown [14,18].

The results obtained by several groups [14,19] also indicate a close colocalization and possible interaction of these proteins in the blood vessels and brains of mice and humans. Lately, it has been shown that misfolded IAPP accelerates Aβ aggregation in vitro and inoculation of misfolded IAPP into mouse brain results in more severe AD pathology and significantly greater memory impairments than in untreated animals [18]. These findings indeed give us a greater understanding of the mechanisms of AD development in T2DM patients.

The cross-seeding model suggests that the aggregation of Aβ is facilitated by the presence of IAPP. However, it is necessary to validate this hypothesis using in vivo models. Such studies will help to determine if there is a physical interaction between the IAPP amyloid and Aβ inside living organisms, providing more compelling evidence for the cross-seeding mechanism of Aβ aggregation.

To study the interaction between amyloidogenic proteins in vivo, we employed the yeast *Saccharomyces cerevisiae* as a model system. Yeast has been widely used for studying mammalian amyloids (see [20,21,22]), including analyses of the interaction between heterologous amyloid aggregates [23,24,25,26] and has provided valuable insights into the interaction between different amyloid aggregates. In previous studies, we have demonstrated a physical interaction between mouse PrP protein aggregates and Aβ peptide in yeast and have identified the regions of PrP responsible for this interaction [23]. Here, we applied a yeast-based model to study the interactions between IAPP, Aβ42, and PrP. Using the FRET (Förster resonance energy transfer) technique, we showed that fluorescently tagged Aβ42 and IAPP co-localize and physically interact. Our data provide evidence for a direct physical interaction between IAPP and Aβ42 aggregates within living cells.

## 2. Results

### 2.1. Human IAPP Fused with YFP Form Amyloid-like Oligomers in Yeast

Previous works, including our studies, showed that fluorophore-tagged mammalian Aβ42 and PrP form amyloid-like aggregates in yeast cells [20,21,27]; these aggregates are similar in properties to aggregates found in the brains of individuals with a disease.

**Figure 1:**
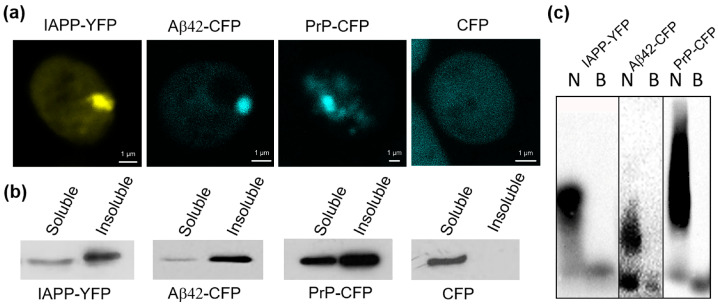
IAPP, Aβ42, and PrP, fused to YFP or CFP, demonstrate amyloid-like properties in yeast *S. cerevisiae*. (**a**) Fluorescent microscopy of cells expressing heterologous proteins: PrP-CFP; Aβ42-CFP; IAPP-YFP; CFP. (**b**) Centrifugation analysis of heterologous proteins in yeast cells. Yeast lysates were centrifuged at 12,000× *g* and thus separated into soluble and insoluble fractions. Proteins were run on SDS-PAGE gel and visualized by Western blotting using anti-GFP antibodies. (**c**) Analysis of IAPP-YFP, Aβ42-CFP, and PrP-CFP aggregates by semi-denaturing agarose gel electrophoresis. Yeast lysates were treated with 3% sarkosyl, after which they were run on agarose gel electrophoresis. Proteins were visualized by Western blotting and reaction to anti-GFP antibodies. B—boiled protein; N—non boiled protein.

Here, we investigated the aggregation of the chimeric IAPP-YFP protein expressed in yeast. We transformed yeast with a multicopy plasmid expressing *IAPP-YFP* under the control of a strong constitutive *P_GPD_* promoter. The resulting transformants were analyzed using confocal microscopy. We showed that the IAPP-YFP protein forms visible foci inside yeast cells (further termed «clumps»). Usually, a cell with microscopically detectable aggregation contained only one clump; in addition, cells with diffuse fluorescence were present (Figure 1a). The frequency of cells with visible clumps was 76 ± 2% of the total number of cells with a fluorescent signal.

Using centrifugation, we also demonstrated that a large portion of the IAPP-YFP protein was found in the precipitated (pellet) fraction, similar to PrP-CFP and Aβ42-CFP, whereas the control CFP protein was present only in the soluble fraction (Figure 1b). Amyloid aggregates are resistant to ionic detergents, such as sodium dodecyl sulfate (SDS) or sodium lauryl sarcosinate (sarkosyl) [21,28,29,30]. Therefore, we treated yeast cell lysates with 3% sarkosyl and fractionated them by semi-denaturing detergent agarose electrophoresis (SDD-AGE), as described previously by Bagriantsev et al. [28]. The data presented in Figure 1c demonstrated that the studied proteins IAPP-YFP, Aβ42-CFP, and PrP-CFP form detergent-resistant polymers in yeast cells. Overall, these data provide evidence that the fluorophore-tagged IAPP forms aggregates with at least some amyloid-like properties in the yeast cell.

### 2.2. The [PIN^+^] Factor Does Not Affect the Aggregation of Heterologous Proteins PrP-CFP, Aβ42-CFP, and IAPP-YFP in the Yeast S. cerevisiae

It is widely recognized that the efficiency of protein aggregation in yeast is affected by a range of internal factors, including the presence of the [*PIN*^+^] prion—an aggregated form of the Rnq1 protein. Previous studies have demonstrated that [*PIN*^+^] is necessary for the formation of aggregates of yeast Sup35 [31,32] and Nup100 [33]. Furthermore, the presence of [*PIN*^+^] can enhance the efficiency of aggregation of the yeast Ure2 protein [34] and nucleoporins [35] and also influence the toxicity of heterologous human huntingtin protein in yeast cells [36].

**Figure 2:**
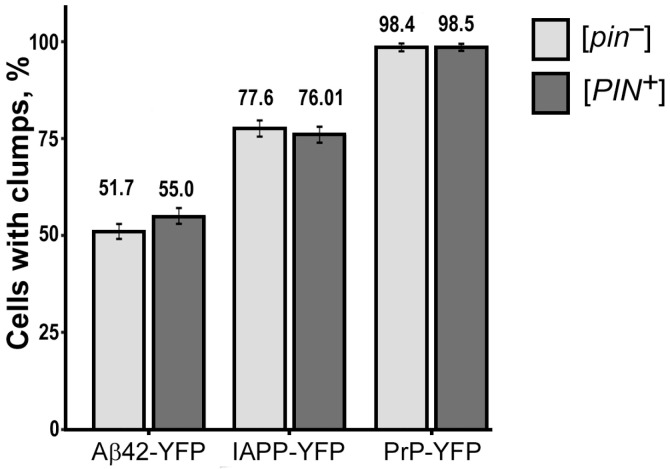
The [*PIN*^+^] factor does not influence the aggregation of PrP-YFP, Aβ42-YFP, IAPP-YFP in yeast. Standard error of the percentage is indicated as error bars. To compare the frequencies of cells with aggregates, Fisher’s exact test was employed. The presented results are based on three separate experiments conducted independently.

In this study, we investigated the impact of the [*PIN*^+^] prion on the aggregation of heterologous proteins (PrP-YFP, Aβ42-YFP, IAPP-YFP) in the yeast *S. cerevisiae*. We used two isogenic strains—BY4742 ([*PIN*^+^]) and AB230 ([*pin*^−^])—that differ in the presence of the [*PIN*^+^] prion. The yeast strains were transformed with plasmids for overproduction of PrP, Aβ42, and IAPP proteins fused with YFP and were then analyzed under a fluorescent microscope (Figure 2).

Our results revealed that all of the investigated proteins formed fluorescent aggregates irrespective of the presence or absence of Rnq1 protein aggregates in the cells. Furthermore, statistical analysis showed no significant difference in the number of aggregates between cells with and without the [*PIN*^+^] prion, indicating that the prion did not play a significant role in the aggregation of the examined heterologous proteins (see Table A1). Based on these results, we can conclude that the [*PIN*^+^] prion does not have a discernible impact on the aggregation behavior of the investigated heterologous proteins in yeast *S. cerevisiae*.

### 2.3. Human IAPP Colocalizes and Physically Interacts with Aβ42 in Yeast Cells

**Figure 3:**
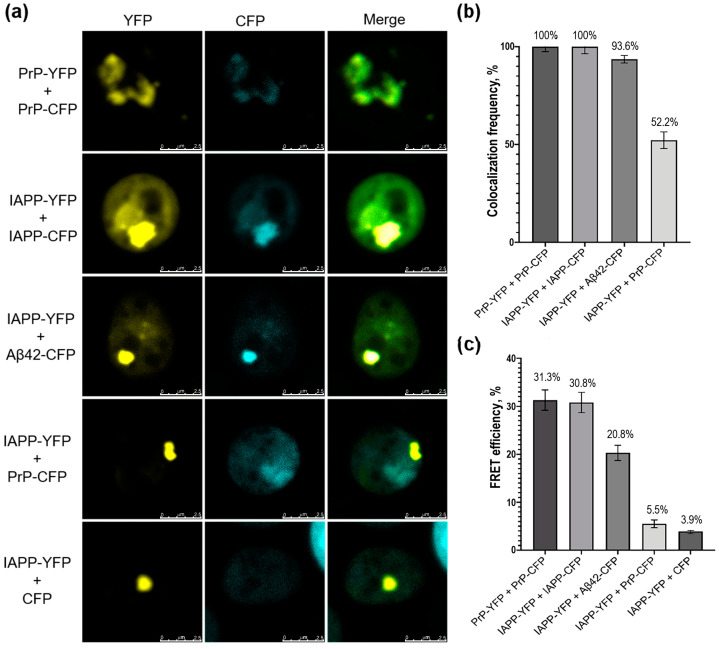
Confocal microscopy of yeast cells co-expressing heterologous proteins. (**a**) (images) and (**b**) (frequencies) colocalization of protein aggregates in yeast. Standard error of percentage is indicated as error bars. (**c**) FRET efficiency for various protein combinations. Standard deviation is indicated as error bars.

To investigate the ability of the IAPP protein to interact with PrP and Aβ42, we co-transformed yeast cells with the following pairwise plasmid combinations: IAPP-YFP and PrP-CFP, or IAPP-YFP and Aβ42-CFP (experimental combinations); IAPP-YFP and IAPP-CFP, or PrP-YFP and PrP-CFP (positive control combinations); and IAPP-YFP and CFP (negative control combination). As a negative control, we used a yeast strain co-producing the amyloidogenic fusion protein IAPP-YFP and a cyan fluorescent protein CFP that does not aggregate by itself and does not induce aggregation of the studied proteins. Respective yeast transformants were analyzed by confocal microscopy (Figure 3).

The results of the confocal microscopy analysis showed that in yeast cells containing microscopically detectable aggregates, there was colocalization of the fluorescent signals in all cells for the positive control combinations (IAPP-YFP and IAPP-CFP, PrP-YFP, and PrP-CFP). In the yeast cells that contained aggregates of IAPP-YFP and Aβ42-CFP, fluorescent signals colocalized with a frequency of 93.6%. In contrast, the clumps of IAPP-YFP and PrP-CFP colocalized only in 52.2% of the cells with both types of aggregates. The data confirmed a possible interaction of the studied chimeric protein pairs in yeast. Similar results were obtained when using the reverse combinations of IAPP-CFP/Aβ42-YFP and IAPP-CFP/PrP-YFP (Figure S1).

**Figure 4:**
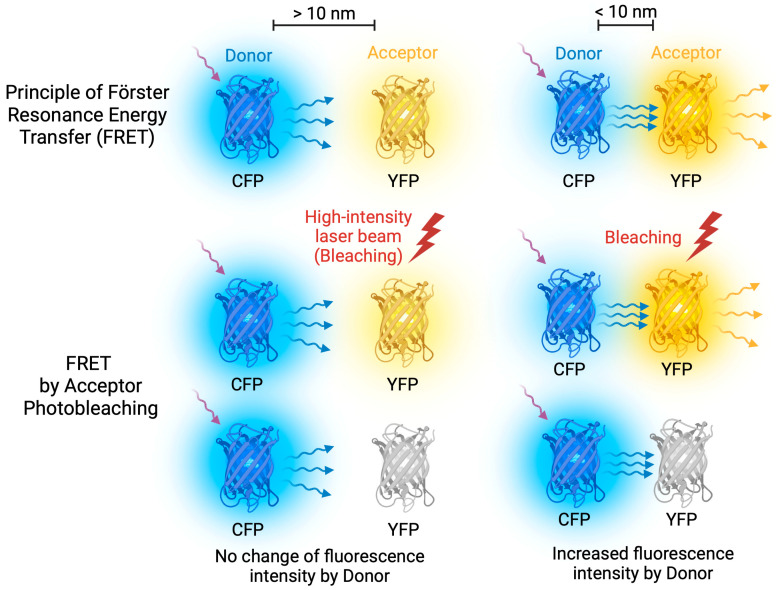
FRET Acceptor Photobleaching. FRET occurs when energy is transferred between donor and acceptor fluorescent molecules that are in close proximity. To achieve optimal FRET, it is important to select fluorescent molecules with overlapping emission and excitation spectra. During FRET Acceptor Photobleaching, a laser beam is used to bleach the acceptor molecule, stopping energy transfer and causing an increase in donor emission. However, if the molecules are too far apart, FRET does not occur.

To further investigate whether colocalized proteins physically interact in yeast cells, we employed the FRET approach [37,38]. FRET is a process of energy transfer between a donor (CFP fusion protein in this study) and an acceptor (YFP fusion protein) molecule at short distances (up to 10 nm), which reflects protein interaction [39,40]. The efficiency of energy transfer was evaluated by comparing the donor fluorescence intensity before and after destroying the acceptor; although the acceptor was photobleached, the donor emitted higher fluorescence in the case of FRET (Figure 4). We used three independent yeast transformants in each case and analyzed at least 100 different cells for each transformant.

The homogenous combinations of PrP-YFP/PrP-CFP and IAPP-YFP/IAPP-CFP) both showed around 31% efficiency of FRET, indicating that these proteins physically interacted in yeast cells. The negative control combination IAPP-YFP/CFP showed only about 4% efficiency of FRET, confirming that there was no physical interaction between these proteins. In a heterogeneous combination of IAPP-YFP/PrP-CFP, a FRET efficiency of 5.5% was detected, which is not statistically different from the negative control and indicated that there was no physical interaction between IAPP and PrP proteins in yeast cells, despite their colocalization. However, for the combination of IAPP-YFP and Aβ42-CFP, a FRET efficiency of 20% was observed, which was significantly different from the negative control and confirmed direct fluorescent resonance energy transfer between the IAPP-YFP and Aβ42-CFP proteins due to resonance. This suggests a physical interaction between IAPP and Aβ42 in a yeast cell. Similar results were obtained when using the reverse combinations of IAPP-CFP/Aβ42-YFP and IAPP-CFP/PrP-YFP (Figure S1).

## 3. Discussion

Numerous studies support a link between T2DM and AD in elderly individuals [41,42,43,44,45,46]. People with diabetes show an increased incidence of cognitive decline and AD [46]. Therefore, patients with T2DM are at a significant risk factor for AD [47]. However, the exact mechanism behind this association is not yet clear. Several hypotheses have been proposed, such as altered insulin signaling, impaired glucose and lipid metabolism (metabolic syndrome), and reduced Aβ clearance capacity [48]. On the other hand, there is a set of evidence to support a direct interaction between IAPP and Aβ. Co-deposition of IAPP and Aβ has been observed in blood vessels and the hippocampus of AD patients’ brains [14]. Based on these findings, it has been suggested that the aggregates of IAPP may contribute to Aβ aggregation and potentially provoke the onset of AD. Arguments in favor of this assumption were obtained in the laboratory of C. Soto [18]. It was shown that synthetic IAPP polymers can cross-seed Aβ42 peptide and enhance its aggregation in vitro. Transgenic mice producing both human proteins (IAPP and Aβ42) exhibit an exacerbated AD-like pathology in comparison to AD-transgenic mice [18]. Moreover, intracerebral injection of misfolded pancreatic IAPP into the hippocampus of AD transgenic mice significantly enhanced AD pathology and memory impairment, compared to untreated animals [18]. Lately, Martinez-Valbuena et al. provided histological evidence that IAPP and Aβ can interact in the human hippocampus and pancreas of patients with AD, as well as in individuals without AD but with a history of T2D [49].

Confirmation of the direct interaction between Aβ and IAPP has been obtained through in vitro experiments (see [50,51]). These studies have shown that Aβ and IAPP can form hetero-oligomers and large aggregate heterocomplexes. In addition, cytotoxicity studies conducted on SH-SY5Y cells demonstrated that co-aggregates of Aβ/IAPP were more toxic to the cells compared to aggregates composed of IAPP or Aβ alone [51]. However, direct evidence of their physical interactions in vivo has been lacking. Recently, Wang and Westermark demonstrated that IAPP and Aβ42 can form heterodimers not only in vitro but also in living Hek293 cells using the bimolecular fluorescence complementation (BiFC) assay [52]. This result supports the hypothesis of direct physical interaction and cross-seeding of these proteins in individuals. However, it remained unclear whether the aggregated version of IAPP can physically interact with Aβ42 and bring it into heteroaggregates. It is also worth noting that a BiFC assay can occasionally yield false positive results. This can be caused by a fluorescent signal of two YFP fragments that are positioned close together (within 7 nm) in a small subcellular compartment, rather than as a result of specific interactions [53,54]. Therefore, additional confirmation by alternative methods was necessary to validate the physical interaction ability of Aβ and IAPP in a living system.

Here, we have applied a yeast model to study the interactions of IAPP with Aβ42 and with PrP. Our findings demonstrate that these proteins form detergent-resistant amyloid-like aggregates in yeast cells. Unlike yeast prions, the aggregation of these proteins does not require the [*PIN*^+^] factor. Using the FRET method, we showed that aggregated IAPP can physically interact with Aβ42 in yeast cells. In contrast, IAPP did not show a physical interaction with PrP, even though these proteins occasionally colocalize (see Figure S2). This indicates that an interaction between IAPP and Aβ is specific for these proteins, rather than reflecting a non-specific association between any amyloids.

The data obtained in our work provide additional support for the hypothesis that toxic polymers of human IAPP formed during T2DM and spread via blood vessels can physically interact with the Aβ42 peptide. This interaction may trigger or enhance Aβ42 aggregation, contributing to the pathogenesis of AD. Understanding the mechanisms of the interaction between IAPP and Aβ proteins could potentially lead to the development of therapies or interventions for both T2DM and AD. By targeting the interaction and aggregation of these proteins, it may be possible to prevent or slow down the progression of both diseases. Additionally, the yeast model can be used to screen for potential drugs or compounds that can inhibit or disrupt the interaction between IAPP and Aβ. This could lead to the development of new treatments for both T2DM and AD.

## 4. Materials and Methods

### 4.1. Plasmids, Strains, Media, and Growth Conditions

*Escherichia coli* strain DH5α [55] was used to host all plasmid construction and maintenance. The yeast *S. cerevisiae* strains BY4742 (*MATα*; *his3Δ-1 leu2Δ-0 lys2Δ-0 ura3Δ-0* [*psi*^−^] [*PIN*^+^]) from Open Biosystems (Huntsville, AL, USA) and AB230 (*MATα*; *his3Δ-1 leu2Δ-0 lys2Δ-0 ura3Δ-0* [*psi*^−^] [*pin*^−^] were used for the study. The AB230 strain is isogenic to the BY4742 strain but does not contain the prion [*PIN*^+^]. This strain was obtained through three passages of the BY4742 strain in YPD media containing 5 mM guanidine hydrochloride (GuHCl). *Saccharomyces cerevisiae* was cultivated at 30 °C.

Standard yeast media were used. The rich organic medium (YPD) contained 1% yeast extract (Helicon, Moscow, Russia), 2% Bacto peptone (BD Biosciences, San Jose, CA, USA), and 2% dextrose (Sigma-Aldrich, St. Louis, MO, USA). A selective synthetic media contained 0.67% yeast nitrogen base (without amino acids) (Sigma-Aldrich, USA), supplemented with essential nutrition (leucine, methionine, tryptophan, adenine, arginine, isoleucine, lysine, phenylalanine, threonine, tyrosine, histidine, valine, and uracil), 0.5% ammonium sulfate (Lenreactiv, St. Petersburg, Russia), and 2% dextrose (Sigma-Aldrich, USA) [56]. The solid media contained 2% agar (US Biologicals, Salem, MA, USA). To study the colocalization and physical interaction between aggregated proteins, yeast co-transformants with the tagged protein coding plasmids were grown in a selective liquid medium lacking leucine and uracil to an optical density (OD) of 0.6–0.8. The OD of yeast cultures was read using a U-2900 spectrophotometer (Hitachi Ltd., Tokyo, Japan)—at a wavelength of 595 nm. Yeast cells were cultivated in a shaking incubator, New Brunswick Innova 43R (Eppendorf, Framingham, MA, USA) at 30 °C, 180 rpm.

All plasmids used in this study were multi-copy shuttle vectors with either *URA3* or *LEU2* markers that can propagate in *E. coli* and yeast *S. cerevisiae* (all the plasmids are listed in Table 1).

The pGPD-Aβ42-CFP (LEU2) plasmid was obtained by inserting the PCR generated product, encoding the human Aβ42 peptide flanked with restriction sites *Sac*II and *Bam*HI into the pGPD-CFP (LEU2) vector digested with the same restriction sites. The sequence encoding human Aβ42 was amplified by PCR from the pGPD-Ab-YFP (LEU2) plasmid using the Aβ-F and Aβ-R primers presented in Table 2.

The plasmids pGPD-IAPP-CFP (LEU2) and pGPD-IAPP-YFP (URA3) encode the human IAPP protein fused in frame with a cyan fluorescent protein (CFP) or yellow fluorescent protein (YFP), respectively. The coding sequence human IAPP corresponds to amino acids 8–37 in mature protein, and its codons were adapted for production in yeast cells. The plasmids were constructed by replacing the *Sac*II-*Bam*HI fragment of pGPD-PrP_23_-YFP (URA3) or pGPD-PrP_23_-CFP (LEU2) with a PCR fragment encoding the human IAPP flanked with restriction sites *Sac*II and *Bam*HI. The coding sequence of IAPP was PCR generated from the plasmid pmCUP1-Sup35NM-IAPP [57] using primers Amy1F and Amy1R (Table 2).

Sanger sequencing was performed to validate the absence of significant mutations in any of the constructed plasmids.

### 4.2. DNA Assays

Plasmid DNA construction was performed according to the standard protocols described by Sambrook et al. [59]. Plasmid extraction and purification from *E. coli* were conducted according to the procedures described by Kachkin et al. [60]. Yeast DNA transformations were performed by a protocol involving lithium acetate treatment and heat shock [61].

### 4.3. Protein Isolation and Analysis

Preparation of cell lysates from yeast and centrifugation were performed according to the protocol described by Chernoff et al. [27] with modifications. The yeast cells were treated with 300 μL of 2 M lithium acetate and then 0.4 M NaOH for 5 min on ice. Cells were resuspended in 100 µL sample buffer (60 mM Tris-HCl pH 6.8, 2% SDS, 10% glycerol, 2% β-mercaptoethanol and 0.01% bromophenol blue) and boiled for 5 min. Then, the cell lysate was centrifuged at 3000× *g* to clear cellular debris. The obtained supernatant was separated into pellets and soluble fractions by centrifugation at 12,000× *g* at 4 °C. The fractions were separated in 12% SDS-PAGE. To detect detergent-resistant IAPP-YFP polymers, the SDD-AGE method was made as described by Bagriantsev et al. [28] with fewer modifications, such as using 0.3% sarkosyl instead of 0.1% SDS. Cell lysates from yeast containing the studied proteins (IAPP-YFP, Aβ42-CFP, PrP-CFP, CFP) were treated with 3% sarcosyl instead of 1% SDS for 10 min at 30 °C. The cell lysates were run on 1.5% agarose gels. Proteins were transferred to an Amersham Hybond P 0.2 PVDF Western blotting membrane (Sigma-Aldrich, USA). Blocking was performed with a 2% Amersham ECL Prime Blocking Reagent (GE Healthcare, Buckinghamshire, UK). To confirm the presence of the proteins of interest in the yeast lysates, specific primary antibodies against the proteins were used: anti-PrP (3F4, Sigma-Aldrich, USA), anti-IAPP (HPA053194, Sigma-Aldrich, USA), anti-GFP (ab13970, Abcam, UK), and anti-Aβ42 (6E10, Abcam, UK) (see Figure S3). Protein detection for the proteins shown in Figure 1 was performed with primary monoclonal antibodies ab13970 against GFP (Abcam, Cambridge, UK) in a 1:7000 dilution and secondary anti-chicken antibodies (ab6877) conjugated to horseradish peroxidase in a 1:200,000 dilution. Chemiluminescent detection was performed using ChemiDoc XRS+ (BioRad, Hercules, CA, USA) with ECL Prime Western Blotting Reagent (Sigma-Aldrich, USA). For normalization of the total protein amount, Coomassie staining was used.

### 4.4. Fluorescence Microscopy

The proteins studied in this work (IAPP, Aβ, and PrP) were fused to one of the fluorescent proteins (CFP or YFP). A confocal laser-scanning microscope Leica TCS SP5 (Leica Microsystems Wetzlar GmbH, Wetzlar, Germany) was used to examine colocalization and the possibility of physical interaction by the acceptor photobleaching FRET (AB FRET) method of the studied proteins. The FRET efficiency was measured as described previously by Rubel et al. [23], using Leica LAS AF X 3.7.2.22383 software (Leica Microsystems Wetzlar GmbH, Germany). CFP, or proteins fused with it in the FRET study acted as a donor (Excitation (Ex) = 458 nm; Emission (Em) = 461–510 nm). YFP or proteins fused with it in the FRET study, acted as an acceptor (Ex = 514 nm; Em = 518–580 nm). Acceptor photobleaching was performed using a 514 nm laser beam at 100% intensity.

Polylysine glass microscope slides from Gerhard Menzel GmbH (Braunschweig, Germany) were used for the FRET experiments. Preliminary yeast cells were washed three times with sterile water, then put onto a glass microscope slide, air-drying, enclosed into Antifade Mounting Medium VECTASHIELD (Vector Laboratories Inc., Newark, CA, USA), and covered with a coverslip (Gerhard Menzel GmbH, Germany).

Confocal microscope data were analyzed using “LAS AF Application Wizard Version 1.7.0” software (Leica Microsystems Wetzlar GmbH, Germany).

### 4.5. Statistical Analysis

To measure colocalization frequencies between IAPP-YFP/PrP-CFP and IAPP-YFP/Aβ42-CFP, 336 cells were analyzed from three independent BY4742 transformants producing the PrP-CFP/IAPP-YFP protein pair and 356 cells producing the Aβ-CFP/IAPP-YFP protein pair in three independent BY4742 transformants. To calculate the colocalization frequencies (Cf) for each pair of proteins, the following equation was used:

$$Cf=\frac{Na}{Ns}\times 100,$$

Na—number of cells with IAPP-YFP aggregates that co-localize with PrP-CFP or Aβ42-CFP; Ns—summary number of cells with both signals analyzed.

To examine the influence of the [*PIN*^+^] prion on the aggregation of heterologous proteins in yeast, we compared the percentage of cells displaying fluorescent focus in both [*PIN*^+^] and [*pin*^−^] strains. Statistical analysis was performed using Fisher’s exact test [62], with *p* ≤ 0.05. Comparisons were performed using Statistica version 13.2 (StatSoft Inc., St. Tulsa, OK, USA) software.

To calculate the effectiveness of the physical interaction between the studied proteins with the FRET method, three independent cultures co-expressing different variants of amyloidogenic proteins fused with CFP/YFP were selected in each case. The FRET efficiency (*FRETeff*) was measured using the software Leica LAS AF X 3.7.2.22383 (Leica Microsystems GmBH, Germany), according to the following equation:

$$FRETeff=\frac{Dpost-Dpre}{Dpost}$$

*Dpre* is the donor fluorescence before photobleaching; *Dpost*—the donor fluorescence after photobleaching.

For the statistical comparison of the FRET efficiencies, we used the Mann–Whitney U-test with the multiple comparisons of z-values. Comparisons were performed using Statistica version 13.2 software; differences with *p* ≤ 0.05.

## 5. Conclusions

The co-aggregation of different amyloidogenic proteins in PMDs suggests an intricate interplay between these proteins in disease pathogenesis. The association between T2DM and AD highlights the need for further investigation of the molecular mechanisms underlying this connection. The yeast-based model used in this study provides a valuable tool for studying the interaction between amyloidogenic proteins and may contribute to a better understanding of the pathogenesis of amyloid-related disorders. In the future, the yeast model can be used to identify the regions and amino acids in IAPP and Aβ that are critically involved in the interaction. In addition, potential factors that can enhance or hinder the interaction and aggregation of these proteins can be identified. Overall, the use of the yeast model in studying the interaction of IAPP and Aβ has the potential to contribute greatly to our understanding of and potential treatment options for both diseases.

## Figures and Tables

**Table 1:** Plasmids used in this study.

Plasmid Name	Promoter/Expression Cassette	Yeast Marker	Source
pmCUP1-Sup35NM-IAPP	*P_CUP1_/Sup35NM-IAPP*	*URA3*	[57]
pGPD-PrP_23_-YFP (URA3)	*P_GPD/_mmPrP(23-231)-YFP*	*URA3*	[23]
pGPD-PrP_23_-CFP (LEU2)	*P_GPD_/mmPrP(23-231)-CFP*	*LEU2*	[23]
pGPD-Aβ42-CFP (LEU2)	*P_GPD_/hsAβ(1-42)-CFP*	*LEU2*	This study
pGPD-YFP (URA3)	*P_GPD_/YFP*	*URA3*	[23]
pGPD-Ab-YFP (LEU2)	*P_GPD_/hsAβ(1-40)-YFP*	*LEU2*	[58]
pGPD-CFP (LEU2)	*P_GPD_/CFP*	*LEU2*	[23]
pGPD-IAPP-YFP (URA3)	*P_GPD_/hsIAPP-YFP*	*URA3*	This study
pGPD-IAPP-CFP (LEU2)	*P_GPD_/hsIAPP-CFP*	*LEU2*	This study

**Table 2:** Primers.

Primer Name	Sequence
Amy1-F	5′-GCGGATCCATGGCCACACAAAGATTGGCTAATTTCC-3′
Amy1-R	5′-TTACCGCGGCACCGCGGTGGCGGCC-3′
Aβ-F	5′-CGGGATCCAATATGGATGCAGAGTTCC-3′
Aβ-R	5′-TCCCCGCGGCGCTATGACAACACC-3′

## Data Availability

Not applicable.

## Supplementary Materials

The following supporting information can be downloaded at: https://www.mdpi.com/article/10.3390/ijms241814122/s1.

## Abbreviations

Aβ	Amyloid beta
AB	acceptor bleaching
AD	Alzheimer’s disease
BiFC	bimolecular fluorescence complementation
CFP	cyan fluorescent protein
Em	Emission
Ex	Excitation
FRET	Förster resonance energy transfer
HD	Huntington’s disease
Htt	Huntingtin
Hs	Homo sapiens
IAPP	islet amyloid polypeptide
OD	optical density
PD	Parkinson’s disease
PMD	protein misfolding disorders
PrP	Prion Protein
SDS	sodium dodecyl sulfate
SDD-AGE	semi-denaturing detergent agarose electrophoresis
T2DM	type 2 diabetes mellitus
TSE	transmissible spongiform encephalopathies
YFP	yellow fluorescent protein
YNB	Yeast nitrogen base
YPD	Yeast Extract–Peptone–Dextrose

## Appendix A

**Table A1:** Number of Cells with Fluorescent Clumps in [*PIN*^+^] and [*pin*^−^] strains. *p* ≤ 0.05 (Fisher’s exact test).

Protein Name	[*PIN*^+^] Status	Total Number of Cells	Number of Cells with Fluorescent Glow	Number of Cells with Fluorescent Clumps	% of Cells with Fluorescent Clumps	*p*-Value
PrP-YFP	[*PIN*^+^]	3939	819	807	98.53 ± 0.42	1
	[*pin*^−^]	3783	739	727	98.38 ± 0.46	
IAPP-YFP	[*PIN*^+^]	4031	429	326	75.99 ± 2.06	0.874
	[*pin*^−^]	3998	397	308	77.58 ± 2.09	
Aβ42-YFP	[*PIN*^+^]	3039	596	328	55.03 ± 2.04	0.534
	[*pin*^−^]	3256	667	345	51.72 ±1.93	
