# Supplementary material for: The Aβ42 Peptide and IAPP Physically Interact in a Yeast-Based Assay
Source: Int J Mol Sci. 2023 Sep 15;24(18):14122. doi: 10.3390/ijms241814122 (PMC10531723; doi:10.3390/ijms241814122)
Supplement: Supplementary file 1 [file ijms-24-14122-s001.zip › ijms-2539532-supplementary.pdf]

Supplementary

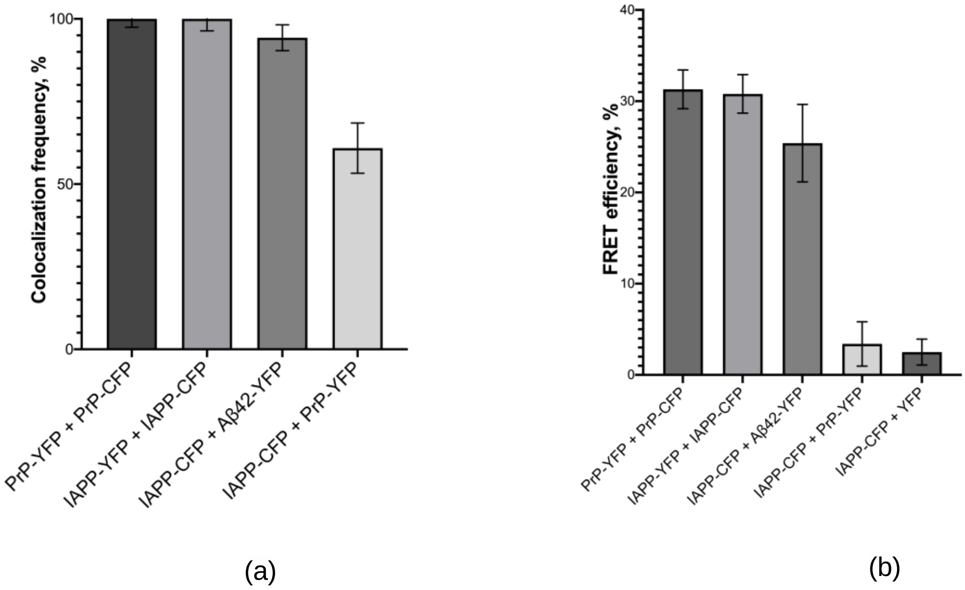

Figure S1. Confocal microscopy of yeast cells co-expressing heterologous proteins. (a) frequencies colocalization of protein aggregates in yeast. Standard error of percentage indicated as error bars. (b) FRET efficiency for various protein combinations. Standard deviation indicated as error bars.

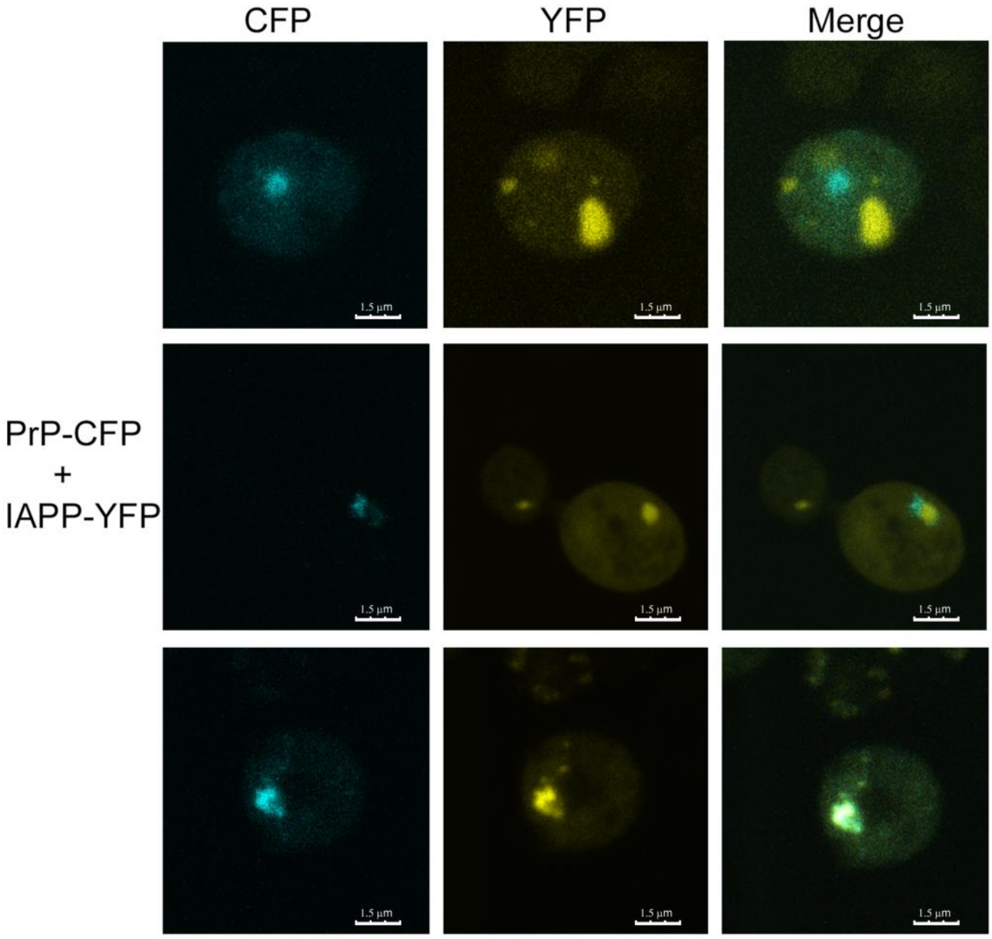

Figure S2. Confocal microscopy of yeast cells co-expressing IAPP-YFP and PrP-CFP proteins. Various localization patterns of IAPP-YFP and PrP-CFP proteins in yeast cells are demonstrated.

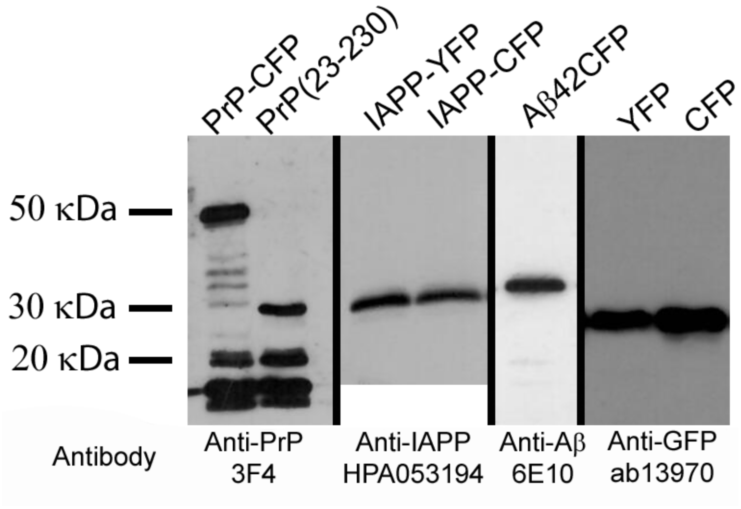

Figure S3. Western blotting analysis of heterologous proteins in yeast cells. Yeast lysates were run on SDS-PAGE gel and visualized by Western blotting using anti-GFP (ab13970, Abcam, UK), anti-PrP (3F4, Sigma-Aldrich, USA), anti-IAPP (HPA053194, Sigma-Aldrich, USA), and anti-Aβ42 (6E10, Abcam, UK) antibodies.
